# The WeThrive App and Its Impact on Adolescents Who Menstruate: Qualitative Study

**DOI:** 10.2196/57936

**Published:** 2024-10-03

**Authors:** Nora MacNeil, Victoria Price, Meghan Pike

**Affiliations:** 1 Medical Science Program Faculty of Science Dalhousie University Halifax, NS Canada; 2 Division of Hematology/Oncology Department of Pediatrics Izaak Walton Killam Health Centre Halifax, NS Canada

**Keywords:** heavy menstrual bleeding, adolescents, menorrhagia, quality of life, mobile applications, mobile health application, mobile phone

## Abstract

**Background:**

Heavy menstrual bleeding (HMB) affects up to 37% of adolescents. Without recognition, HMB can lead to other medical conditions resulting in diminished health-related quality of life. WeThrive, a new mobile health (mHealth) app, implements the pictorial bleeding assessment chart to identify HMB, and the adolescent Menstrual Bleeding Questionnaire to measure the effects of HMB on adolescents’ health-related quality of life. If HMB is identified, WeThrive will connect users to local clinics for further assessment of their menstrual bleeding with a health care provider.

**Objective:**

This study aimed to describe adolescents’ experiences using WeThrive app.

**Methods:**

This qualitative study was approved by the local Research Ethics Board in Halifax, Nova Scotia, and informed consent was provided by all participants. Individual semistructured interviews were held via videoconference with adolescents younger than 18 years, who had at least 1 menstrual period and had used WeThrive at least once. Interview transcripts were thematically analyzed by 2 investigators (MP and NMN) independently, and the κ statistic was calculated to determine the strength of correlation in themes.

**Results:**

Five adolescents (mean age 15.5, range 13-18 years), participated in the interviews. All participants stated that WeThrive helps them better understand their menstrual periods by predicting period onset, recognizing menstrual symptoms, and identifying HMB. Four themes were identified: (1) the importance of visual features and usability, (2) newly obtained knowledge using WeThrive, (3) feature use depends on menstrual health, and (4) trustworthiness. There was substantial agreement on the identified themes (κ=0.73).

**Conclusions:**

WeThrive is visually appealing, and trustworthy, and helps users better understand their menstrual periods, including identifying HMB. By identifying HMB early, WeThrive has the potential to improve the recognition of bleeding disorders and iron deficiency in adolescents. WeThrive is a useful tool to help adolescents better understand their menstrual periods.

## Introduction

Adolescents frequently have difficulty assessing the regularity of their menstrual cycles as they may be unaccustomed to what is considered normal [1]. Adolescents are often hesitant to discuss their menstrual periods, which may lead to abnormal bleeding patterns going undiagnosed [1]. Heavy menstrual bleeding (HMB) is defined as a blood loss of >80 mL per menstrual cycle and affects up to 37% of adolescents [2]. Health-related quality of life (HRQoL) refers to the impact that illness, health, and treatments have on an individual’s quality of life [3]. Many studies have demonstrated that HMB can decrease HRQoL by limiting a woman’s social, material, physical, and emotional quality of life [4,5]. It is extremely important to identify HMB early, to identify underlying causes (eg, an underlying bleeding disorder), which can exacerbate the decline in HRQoL. However, quantifying menstrual blood loss is impractical for many women.

The adolescent Menstrual Bleeding Questionnaire (aMBQ), a 25-item questionnaire developed by Pike et al [2], is a valid and reliable tool to measure the impact of HMB on an adolescent’s HRQoL. The aMBQ identified a direct relationship between HMB and HRQoL [4]. The aMBQ is scored on a scale of 0-77, with higher scores indicating a worse HRQoL and a score of >30 identifying HMB with a specificity of 84.4% [2]. The pictorial bleeding assessment chart (PBAC) is a validated tool used to track the saturation of menstrual products, with a score of >100 denoting HMB [5]. Clinical experience indicates that adolescents are hesitant to fill out the aMBQ or the PBAC on paper. Given this, physician investigators launched a new mobile health (mHealth) app, WeThrive, which is specialized in its design for use by adolescents and includes the aMBQ and the PBAC to identify HMB. WeThrive incorporates a calendar to track users’ menstrual cycle, period prediction, symptom tracking, and answers to frequently asked questions regarding menstruation. If HMB is identified, WeThrive will connect users to local clinics for further assessment of their menstrual bleeding with a health care provider. The primary objective of this study was to describe adolescents’ experiences using WeThrive app.

## Methods

### Ethical Considerations

This was a qualitative study conducted at the Izaak Walton Killam (IWK) Health Centre in Halifax, Nova Scotia. The study included individual semistructured interviews and thematic analysis. This study was approved by the IWK Research Ethics Board (1028441). Informed consent was provided by all participants before interview completion. All study data are anonymous and deidentified. All information provided by participants was anonymized, including names, and any potential identifiers such as the names of schools and siblings’ names. Each participant was assigned a unique number used as a reference to replace names. Each participant was provided a US $7.40 gift card as compensation for their time.

### Recruitment of Participants

Adolescents younger than 18 years, who had at least 1 menstrual period and used WeThrive at least once, were invited to participate in an interview. Study participants were known to the physician investigator through the pediatric hematology or oncology clinic; however, the physician investigator was not involved in the participants’ medical care at the time of interview completion. The student interviewer did not have a relationship established before interview completion. Student investigator explained the objective of the interview, specifically that investigators wanted participants to answer the guiding questions honestly, and that there were no right or wrong answers to the prompted questions. Participants were encouraged to share any thoughts they had regarding their experiences using WeThrive. Participants were recruited via a social media advertisement posted on WeThrive accounts (Instagram: @we_thrive_app, Twitter: @for.teens.period) and from the pediatric hematology or oncology clinic. This social media post was used to invite interested adolescents to participate in the study, given they meet the inclusion criteria. If interested, individuals contacted physician investigators via email. Physician investigators were not involved in any participants’ medical care at the time of interview participation. Sample size was determined based on the validity of the interviews. Two individuals recruited from the social media posts were selected to participate in the study; however, these interviews were discarded due to the stipulation that 1 individual had participated in 2 interviews on 2 separate occasions under different names. After discarding 2 interviews, the eligible participant sample size was 5. This decision was approved by members of the local Research Ethics Board.

### Interview Process

Interviews were conducted via videoconference by 2 investigators (MP and NMN), scheduled as 45-minute interviews. One physician investigator (pediatric hematologist and oncologist) and 1 undergraduate student investigator were present for the interviews. Interviews were conducted by the student investigator while being supervised by a physician investigator. Both investigators are female and have completed the Tri-Council Policy Statement: Ethical Conduct for Research Involving Humans. Student investigator explained the objective of the interview, specifically that investigators wanted participants to answer the guiding questions honestly, and that there were no right or wrong answers to the prompted questions. Participants were encouraged to share any thoughts they had regarding their experiences using WeThrive. Interviews were recorded and later transcribed. No repeat interviews were carried out. The interviews were semistructured with guiding questions as outlined in Multimedia Appendix 1. During the interviews, field notes were collected, including observations of participants’ behaviors, speech, and overall engagement. Sampling was terminated when no new information was provided from interviewing additional participants. Researchers felt that sampling redundancy was achieved. Information power was used as a pragmatic guiding principle as suggested by Vasileiou et al [6]. Since this study included participants with HMB and symptoms related to their menstrual periods, researchers are comfortable with the sample diversity within a sample size of 5. Interview transcripts were not returned to the study participants.

### Data Analysis

Two investigators (MP and NMN) independently coded the anonymized transcriptions and field notes. Responses were coded manually using color coordination between similar words and ideas. This information was then grouped into themes, which emerged as similarities between interviews, indicating sampling redundancy. Investigators determined themes independently, then a meeting was held to determine whether their codes and themes aligned, and any discrepancies were adjudicated by a third independent reviewer (VP). Themes were derived from data. The κ statistic was calculated to determine the strength of correlation in themes. Participants did not provide feedback on the investigators’ findings.

## Results

### Overview

Interviews were held with 7 participants. We excluded information from 2 participants as we could not confirm whether they met the inclusion criteria. Therefore, data were analyzed from 5 participants. The mean age of participants was 15.5 (range 13-18) years. Four of the 5 participants were White, and 1 participant was African American. All participants were enrolled in high school and have their own smartphone. The mean length of interviews was 18 (range 11-25) minutes. Participants used WeThrive for a mean of 5.8 (range 3-12) months each before interview participation (at least once per month; for the week before and during their menstrual period). Participants spoke freely, had their cameras on, and were smiling and engaged. All interviews were shorter than anticipated, potentially due to the comfort levels of the participants.

**Figure 1:**
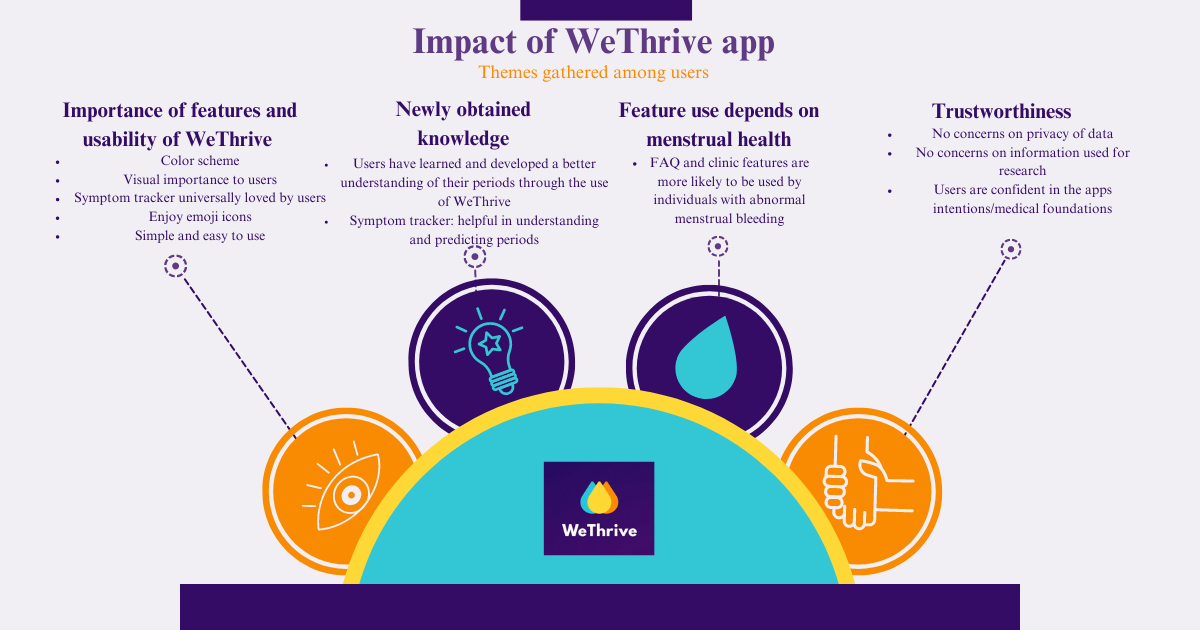
Four themes were identified from individual semistructured interviews held via videoconference for a qualitative study looking to describe adolescents’ experiences using WeThrive app. Interviews were conducted on 5 adolescents younger than 18 years, who have had at least 1 menstrual period and have used WeThrive app on at least 1 occasion. FAQ: frequently asked question.

There was substantial agreement on the identified themes (κ=0.73). The themes are shown in Figure 1 and include (1) the importance of visual features and usability, (2) newly obtained knowledge using WeThrive, (3) feature use depends on menstrual health, and (4) trustworthiness.

#### Theme 1: The Importance of Features and Usability of WeThrive

Four (80%) participants enjoyed the color scheme and the overall appearance of WeThrive*,* stating that they liked “the concept of not using classic colours [like pink and red],” while 1 (20%) participant stated a preference for a lighter color scheme. Moreover, 40% (4/10) of total suggestions for improvement of WeThrive pertained to its visual appearance. Overall, the number of comments regarding the app’s appearance illustrates the importance of visual features. Participants commented on the simple layout and design of WeThrive. One participant described WeThrive as “perfect” because “it’s simple and easy to use”; this contributed to their decision to use the app. Another participant shared that she “likes how WeThrive is easy to navigate.” All participants used WeThrive regularly, at least once per month, within the week before and during their menstrual period. The symptom tracker was valued as the favorite and most frequently used feature of WeThrive*.* All 5 participants enjoyed the emoji choices for picking symptoms (Multimedia Appendix 2). The symptom tracker was essential for participants to obtain new knowledge about their menstrual periods (see Theme 2).

#### Theme 2: Newly Obtained Knowledge

All 5 (100%) participants learned something new about menstrual periods by using WeThrive. Participants learned the possible symptoms and emotions they could experience during their menstrual period. Two (40%) participants found that the symptom tracker helped them understand when their menstrual period would start. WeThrive identified HMB in 1 (20%) participant. This participant was then able to share her PBAC score with her physician. Another participant concluded with “I think it’s a very good app to understand your period better …. I think it would help a lot of people.”

#### Theme 3: Feature Use Depends on Menstrual Health

The frequently asked questions were used by only those participants who had symptoms related to their period (n=2, 40%) or HMB (n=1, 20%). The participant who identified HMB using WeThrive accessed the “clinics features” and was able to share her PBAC score with a physician. One participant stated, “I haven’t used [the Clinic lists] but it’s good that it’s there for those who need it.”

#### Theme 4: Trustworthiness

All 5 participants agreed that the app is clear about what information will be used for research and trusted that their data are securely stored. One participant shared that she appreciated the medical foundations of the app; she felt that WeThrive was developed by trustworthy sources. All participants universally agreed that WeThrive’s privacy policy and research statements were clear upon app launch, demonstrating their trust in the use of the app and the app’s intentions.

### Suggestions for Improvement

Participants shared several suggestions for improving WeThrive. Participants felt that there should be a broader range of emotions available to log in to the symptom tracker. One (20%) participant felt that there should be an emoticon representing irritability. In addition, one (20%) participant felt that it may be useful to log the severity of symptoms experienced (low, medium, and high). Participants felt that providing notifications or animations to remind users to log the last day of their period would be helpful. Finally, participants felt that incorporating the calendar into the opening screen or implementing a dashboard may improve the app’s organization.

## Discussion

### Principal Findings

The results of this study describe adolescents’ experiences using WeThrive app and illustrate the impact of WeThrive on adolescents who menstruate. Adolescents report that WeThrive is visually appealing, easy to use, trustworthy, and contains a very helpful emoji-based symptom tracker. The use of WeThrive facilitated further understanding of menstrual periods, for example, by identifying symptoms predictive of menstrual cycles. WeThrive successfully identified HMB in 1 participant, who was able to use the app’s “clinic feature” to connect to a physician. This participant may have otherwise been unaware of HMB. By identifying HMB early, WeThrive has the potential to improve the recognition of bleeding disorders and iron deficiency in adolescents.

Our study confirms previously published evidence that the visual appearance of an mHealth app is important to adolescents [7,8]. Many menstrual tracking apps are designed to be pink in accordance with gender stereotypes for “girls,” which may present a barrier for transgender youth who wish to use a menstrual tracking app. Research has concluded that transgender youth face barriers to sexual and reproductive health care [9]. Thus, WeThrive was developed as a gender-neutral mHealth app as researchers recognize that not every adolescent who menstruates identifies as a female.

Jeminiwa et al [10] indicated that adolescents are more drawn to mHealth apps that are established by trustworthy sources. Our data support this concept and underscore the importance of the associations between mHealth apps, access to evidence-based information, and association with medical professionals.

Research has previously identified that symptom tracking facilitates further understanding of menstrual periods [11]. Specifically, individuals who experience abnormal menstrual periods develop patterns of their irregularities via tracking [11]. In this study, WeThrive helped users better understand their menstrual periods by identifying symptoms predictive of menstrual cycles, and WeThrive accurately identified HMB in 1 participant. Research also suggests that the more educated adolescents are regarding their menstrual periods, the less anxiety they experience when they occur [12]. Many adolescents are drawn to tracking their menstrual periods in mHealth apps because they are a private, confidential way to learn about their menstrual health and better understand new developments in their bodies [13]. Menstrual charting associated with mHealth apps encourages positivity in adolescents who menstruate. This menstrual charting can then be shared with clinicians, which has the potential to increase the identification of abnormal menstrual periods and improve the recognition of bleeding disorders and iron deficiency in adolescents [1].

Participants commented on ways to improve the app, which developers should keep in mind when designing mHealth apps for adolescents. Participants suggested that notifications and reminders are preferred when the app requires logging information (eg, logging the last day of a period). In addition, if a symptom log is included in an app, it should include a broad range of symptoms and offer the ability of an adolescent to “rate” symptoms based on severity. Participants also suggested that app content should be organized via a dashboard upon launch for easy access. Our study findings add to a previously published systematic review examining the preferred features of mHealth apps in adolescents [10].

Researchers acknowledge a limitation of the study in the potential for selection bias and attempt to mitigate this risk by recruiting participants through multiple strategies. All participants received a standardized introductory message. Physician investigators were not involved in any participants’ medical care at the time of interview participation. Researchers do not believe that selection bias is relevant due to the respondent’s willingness to provide constructive criticism. Researchers also acknowledged the limitations of the small cohort but felt that themes were common among participants and that interviewing more adolescents likely would not have added new information. In addition, the presence of one of the app developers during the interviews may have contributed to the hesitancy of the interview participants to provide negative feedback; however, most participants remained open to providing constructive criticism to further the development of the app. This may reflect the fact that in building and designing WeThrive, app developers incorporated the views and opinions of a group of adolescents to optimize the target audience’s (adolescents’) experience in using WeThrive*.*

### Conclusions

WeThrive is a visually appealing, easy-to-use, and trustworthy mHealth app that improves adolescents’ understanding of menstrual periods and offers unique features for those seeking more information or clinical care. Users were most impacted by WeThrive’s assistance in recognizing symptoms, which could help them predict their menstrual periods. By successfully identifying HMB and connecting users to health care professionals for further evaluation, WeThrive has the potential to improve the recognition of bleeding disorders and iron deficiency within the adolescent population.

## Appendix

Multimedia Appendix 1 Guiding questions asked during individual semistructured interviews held via videoconference. Questions were used as a guide in a qualitative study with the aim to describe adolescents’ experiences using WeThrive app.

Multimedia Appendix 2 Emoji icon choices for period symptom and emotion tracking featured in WeThrive app, a new mHealth app specialized in its design for use by adolescents that includes the adolescent Menstrual Bleeding Questionnaire and pictorial bleeding assessment chart to identify heavy menstrual bleeding.

## Abbreviations

aMBQ: adolescent Menstrual Bleeding Questionnaire
HMB: heavy menstrual bleeding
HRQoL: health-related quality of life
mHealth: mobile health
PBAC: pictorial bleeding assessment chart

## Data Availability

The datasets generated during or analyzed during this study are available from the corresponding author upon reasonable request.
