# Supplementary material for: The WeThrive App and Its Impact on Adolescents Who Menstruate: Qualitative Study
Source: JMIR Form Res. 2024 Oct 3;8:e57936. doi: 10.2196/57936 (PMC11487203; doi:10.2196/57936)
Supplement: Multimedia Appendix 1 [file formative_v8i1e57936_app1.docx]

| Engagement questions  • How did you hear about *WeThrive*?  • How long have you been using *WeThrive*?   Exploration questions  • What features in the app do you like/use the most? Which do you like the least?  • How often do you use *WeThrive*?  • Are there any other questions that you would like to see addressed in the Frequently Asked Questions (FAQ) section?  • How frequently do you use the Flow Check feature (aMBQ)?  • How frequently do you look at your Period Summary (PBAC)?  • Have you used the app to communicate with a healthcare provider or parent/caregiver? Can you give us an example?  • Did using the app identify something about your period that you did not know before?   Exit question  • Is there anything else you would like to add about the app, any comments, suggestions, or general feedback? |
| --- |
