# Supplementary figures and images for: The WeThrive App and Its Impact on Adolescents Who Menstruate: Qualitative Study
Source: JMIR Form Res. 2024 Oct 3;8:e57936. doi: 10.2196/57936 (PMC11487203; doi:10.2196/57936)

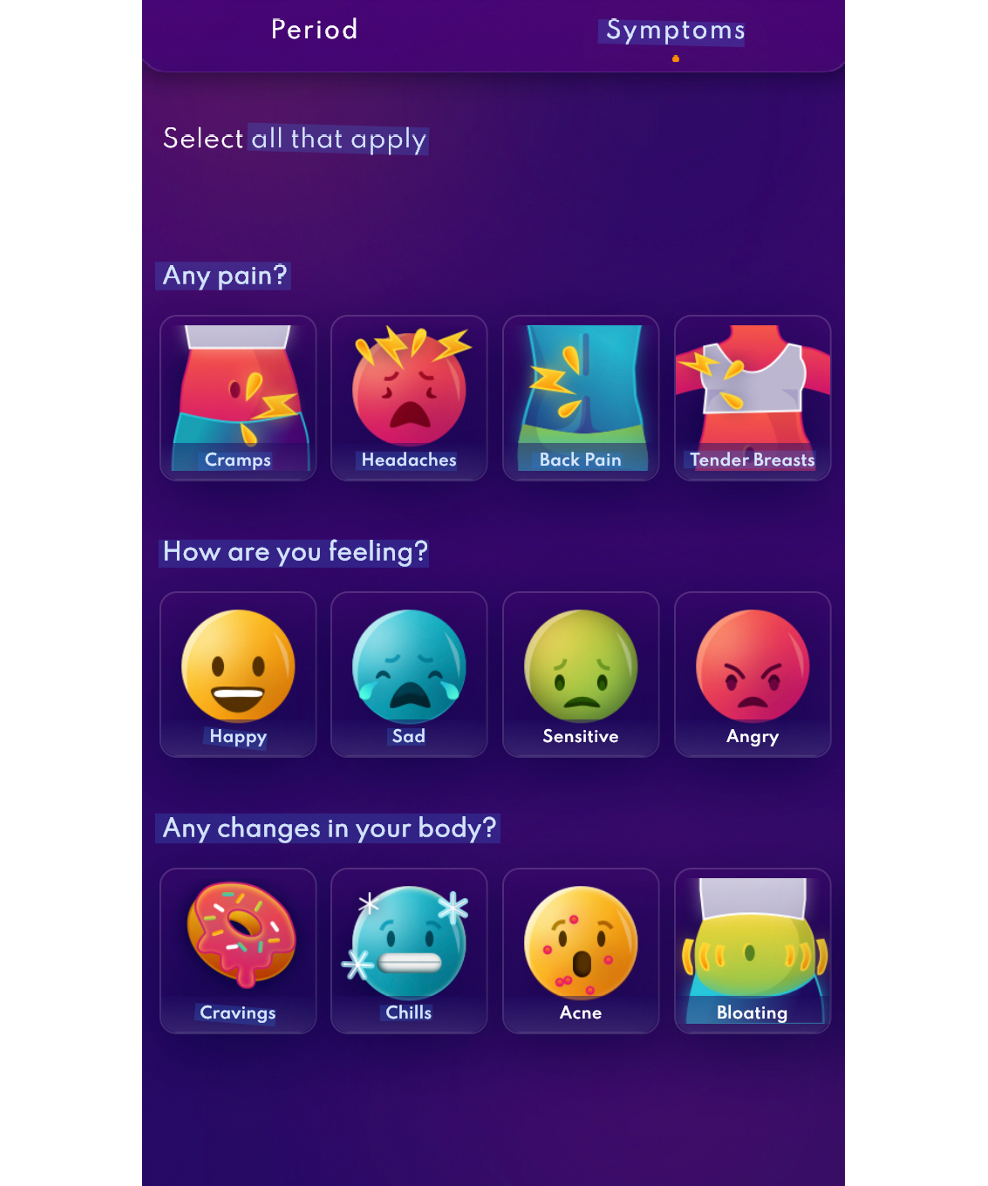

Supplement: Multimedia Appendix 2 [file formative_v8i1e57936_app2.png]
